# Optimizing Femoral Access in Emergency EVAR with a Decision-Making Algorithm

**DOI:** 10.3390/life14091113

**Published:** 2024-09-04

**Authors:** Domenico Mirabella, Salvatore Bruno, Manfredi Agostino La Marca, Ettore Dinoto, Edoardo Rodriquenz, Andrea Miccichè, Felice Pecoraro

**Affiliations:** 1 Vascular Surgery Unit, AOUP Policlinico “P. Giaccone”, 90127 Palermo, Italy; dmirabella@live.it (D.M.); salvatorebruno93@gmail.com (S.B.); manfredi.a.lamarca@gmail.com (M.A.L.M.); edoardo.rod95@gmail.com (E.R.); andrea.micciche01@gmail.com (A.M.); felice.pecoraro@unipa.it (F.P.); 2 Department of Surgical, Oncological and Oral Sciences, University of Palermo, 90133 Palermo, Italy

**Keywords:** femoral access, percutaneous access, rAAA, EVAR, surgical conversion

## Abstract

Endovascular aneurysm repair (EVAR) has become the preferred approach over open repair for abdominal aortic aneurysms (AAAs) due to its minimally invasive nature. The common femoral artery (CFA) is the main access vessel for EVAR, with both surgical exposure and percutaneous access being utilized. However, in emergent cases, percutaneous access can be challenging and may result in complications such as bleeding or dissection thrombosis, leading to the need for surgical conversion. This study aimed to share experiences in implementing a decision-making algorithm to reduce surgical conversions due to percutaneous access failures. A total of 74 aortic patients treated with EVAR in emergency settings were included in this retrospective study. This study focused on various outcomes such as perioperative mortality, morbidity, procedure time, surgical exposure time, and surgical conversion rate. After the implementation of the decision-making algorithm, decreases in surgical conversions and operating time were observed. Percutaneous access was found to be more challenging in cases with specific anatomical characteristics of the CFA, such as severe atherosclerosis or smaller vessel diameter. This study highlighted the importance of carefully assessing patient anatomical features and utilizing a decision-making algorithm to optimize outcomes in EVAR procedures. Further research is needed to continue improving practices for managing aortic aneurysms and reducing complications in femoral artery access approaches.

## 1. Introduction

**Figure 1:**
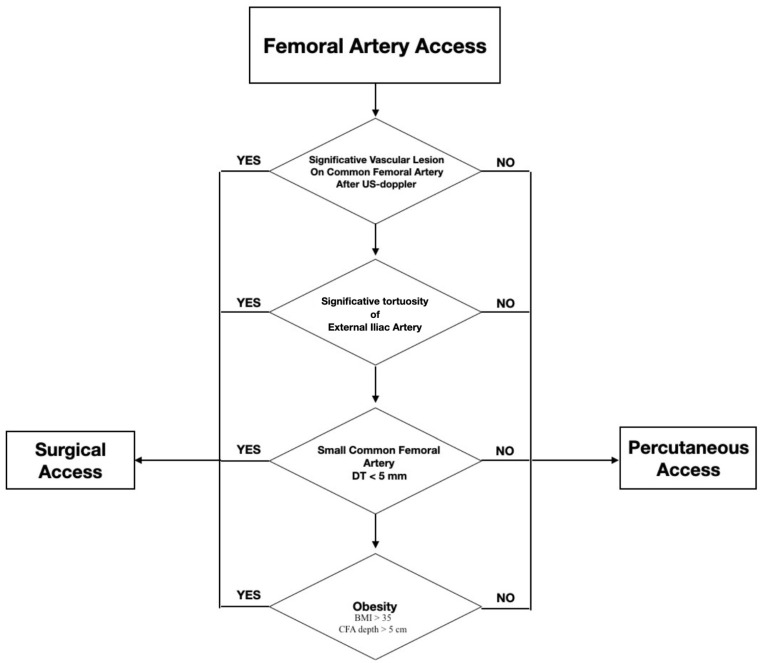
Flowchart showing the decision-making algorithm employed in an AAA emergency.

Endovascular aneurysm repair has become a widely used and effective treatment option for patients with abdominal aortic aneurysms. The minimally invasive nature of EVAR has surpassed open repair in AAAs, resulting in decreased hospitalization time and fewer local complications [1,2]. One of the key aspects of a successful EVAR procedure is the proper selection and optimization of femoral access. The CFA is the main vessel used for access during EVAR, either through surgical exposure or percutaneous access, where suture-mediated closure devices are placed at the beginning of the procedure before the insertion of high-caliber sheaths [3,4]. The percutaneous technique is a simple approach, but in our experience during emergent treatment, it may not be as easy or free of consequences. However, there is no consensus in vascular surgery community regarding the choice of access in endovascular aortic repair [5]. In emergency EVAR cases, where time is limited and the patient may be hemodynamically unstable, achieving optimal femoral access becomes even more critical. In fact, the need for rapid access to hard or deep arteries can more easily lead to bleeding or dissection thrombosis, necessitating an urgent surgical cutdown. Complications at the access site can prolong operating time and increase risks for the patient. In an emergency, acting quickly and making well-informed decisions is crucial in order to minimize potential complications. There are limited data on the best access type for treating ruptured AAAs (rAAAs) with EVAR. The purpose of this study is to share our experience in implementing a decision-making algorithm that has effectively decreased the occurrence of surgical conversions resulting from percutaneous access failures (Figure 1). Here, the different factors that need to be considered when selecting the optimal access site, as well as the potential complications that can arise if proper access is not achieved, are reported.

## 2. Materials and Methods

This study was retrospective and included a total of 74 aortic patients treated in emergencies with EVAR from February 2017 to February 2023. All patients were collected and inserted into standardized piloted forms. All the included patients gave informed consent to be included in this study, anonymous data collection, and analysis. This study was performed in agreement with the Declaration of Helsinki, and the STROBE guidelines for reporting observational studies were followed [6]. The measured outcomes of perioperative mortality and morbidity, time of the procedure, time of surgical exposition of the femoral artery, and surgical conversion rate were all registered. Additional maneuvers on the CFA, such as endoarterectomy, stenting, or angioplasty, were reported. Patients treated with a surgical approach during previous procedures, thoracic aortic treatments, iliac access, or bypasses involving the CFA were excluded. The preoperative visit assessment consisted of a duplex ultrasound (DUS) and computed tomography angiography (CTA) as part of the preoperative evaluations of aortic disease. The collected variables included demographics, comorbidities, clinical data, preoperative imaging studies, procedure details, types of intervention, types of anesthesia, blood transfusions, medical therapies, and lengths of stay. Renal function was estimated using the Chronic Kidney Disease Epidemiology Collaboration (CKD-EPI) [7]. The patients were divided into two groups: before February 2021 (group A) and after the same date (group B). The division stemmed from the implementation of a decision-making algorithm for access starting in February 2021, aimed at reducing the number of complications. The collected data were retrospectively analyzed in October 2023. The early outcomes measured included in-hospital mortality, morbidity, symptom recurrence, and bleeding. The late outcomes included mortality and symptom recurrence. A correlation analysis of age, comorbidities, type of treatment, type of femoral approach, blood transfusion, reinterventions, number of surgical conversions of access, and hospital stays with complications and death was performed. Clinical follow-up consisted of a clinical examination; a CT scan at 2 months; and a DUS at 1 month, after 6 months, and every 12 months thereafter. The median follow-up was 28.77 (mean: 24; r: 12–52; standard deviation [SD]: 16.43) months. For statistical analysis, means and SDs or medians and ranges were reported for parametric data; absolute values and percentages were reported for non-parametric data. Differences in preoperative and postoperative outcomes were assessed using the Student *t*-test. A bivariate test was used to assess relationship significance for correlation analysis. Statistical significance was considered at *p* < 0.05. These values were log-transformed for discrete skewness. We tested for linearity using a test for linear trends across the quartiles. Statistical analysis was performed using SPSS 16.0 (SPSS Inc., Chicago, IL, USA).

### 2.1. Technique—Surgical Approach

**Figure 2:**
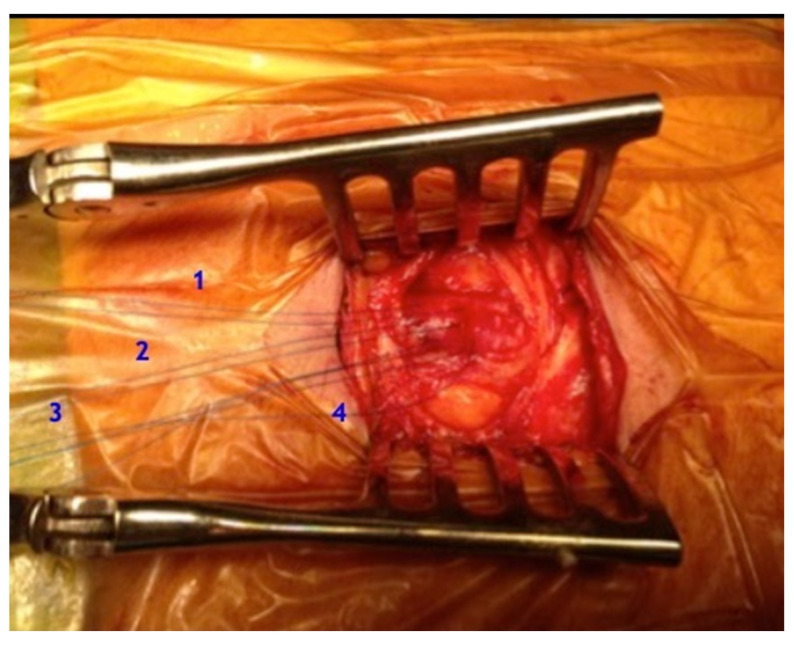
CFA surgical access with Surgiclose technique. The four sutures are highlighted by the numbers 1 to 4.

In our unit, the surgical approach consisted of the Surgiclose technique [8]. The anterior surface of the CFA is exposed through a small longitudinal incision below the inguinal ligament. Four preliminary 5-0 polypropylene (RB needle) transmural single sutures are placed in the horizontal axis at a distance of 1 to 2 mm [9] (Figure 2). The vessel is then punctured with a femoral angiographic needle in the midline between the 4 sutures, and the corresponding sheaths are inserted over the wire [10]. At the end of the procedure, the sheath and wire are removed, and all 4 sutures are pulled tight by an assistant while the surgeon ties all the sutures sequentially [11].

### 2.2. Technique—Percutaneous Approach

**Figure 3:**
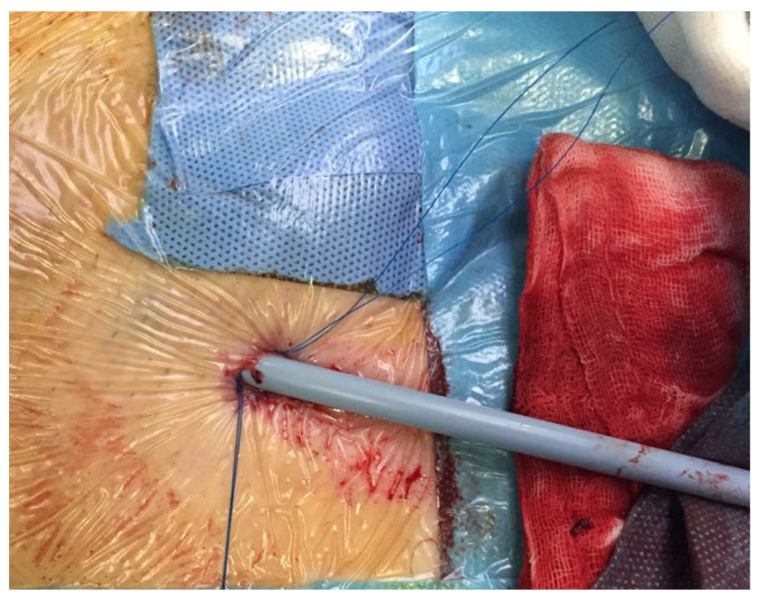
Intraoperative application of Perclose ProGlide.

There are two main percutaneous closure systems: suture-mediated closure devices like Perclose ProGlide devices (Abbott Vascular, Chicago, CA, USA) and plug-based vascular closure devices such as MANTA (Teleflex, Wayne, PA, USA). The ProGlide deploys a suture on each side of the arteriotomy site. The device is inserted over a wire inside the vascular lumen, and the arteriotomy is closed by tightening the suture loop [12] (Figure 3).

**Figure 4:**
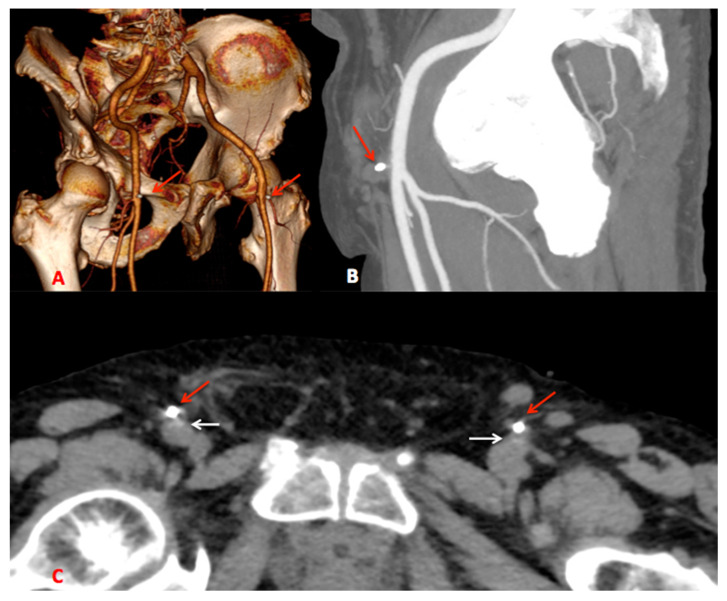
TC images with application of the MANTA device: (**A**) 3D reconstruction with the external fragment of the indicated device highlighted by red arrows; (**B**) sagittal reconstruction with the external fragment indicated by red arrows; and (**C**) basal TC with the external fragment of the reported device indicated by red arrows and the internal collagen plug indicated by white arrows.

The MANTA device includes an absorbable footplate and a collagen plug that are deployed at the end of the procedure to internally cover and seal the arteriotomy with collagen (Figure 4).

### 2.3. Algorithm

This model was applied to the second group of patients included in this study. The algorithm applied was a simple decision-making process based on evaluating the most frequent causes of hemostasis system failures. Personal experience can lead to underestimating the specific anatomical characteristics of a patient. Our algorithm focused on evaluating the CFA both after DUS and after CT. Severe atherosclerosis with calcifications (arterial calcifications were categorized as none; mild: <25% circumference; moderate: 25–50%; or severe: >50%), severe tortuosity of the iliac axes (ITI) (ratio of the center lumen-line distance between the common femoral artery and the aortic bifurcation and the straight-line distance between the common femoral artery and the aortic bifurcation: >1.6), a transverse diameter of the common femoral artery of less than 5 mm, and moderate to severe obesity (BMI: >35) with a CFA depth of greater than 5 cm are conditions that predispose to bleeding from percutaneous access.

## 3. Results

This study included 74 patients. The first group, prior to the algorithm implementation, was composed of 46 patients (group A), and the second group of 28 (group B). The mean age was 74.88 (IQR: 53–90) years, and eight patients (11%) were female. Nonanatomic patient variables and medical therapy with the related grading system are reported in Table 1 and Table 2, respectively.

**Figure 5:**
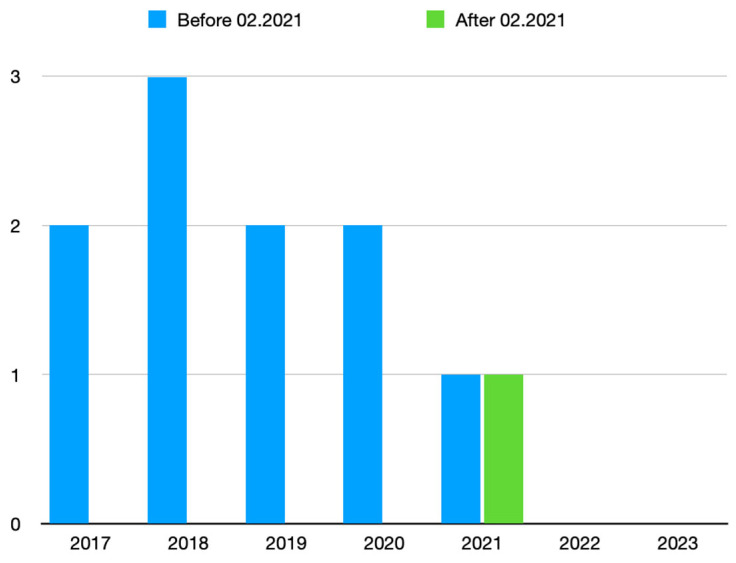
Case trend of surgical cutdown before and after the application of the algorithm.

Of the 74 patients included, 72 (97.4%) were diagnosed with infrarenal aortic aneurysms, 1 (1.3%) with a juxtarenal aortic aneurysm, and 1 (1.3%) with a pararenal aortic aneurysm. All cases were treated as emergencies in patients with rAAAs (52–70.3%) or abdominal pain (22–29.7%). All procedures had a bilateral approach and were reordered with 148 accesses, 11 patients (14.9%) had one femoral artery approached percutaneously and one femoral artery treated with surgical exposure, and 34 patients (46%) were subjected to direct bilateral surgical access with the Surgiclose technique. In 69 (46.6%) with percutaneous access, 11 had surgical cutdowns for bleeding (9) or femoral dissection with thrombosis (2) (Figure 5). The surgical conversions for bleeding were necessary at the start of the procedure after arterial catheterization and ultrasound checks that showed hematomas in the CFA in seven patients and two cases after removal of the sheath, while two conversions for thrombosis/dissection became necessary for limb ischemia (Table 3).

Of the 11 conversions, 8 involved the ipsilateral side of the main body, 3 involved the contralateral leg, and all cases occurred before the algorithm was applied. In all seven cases of conversion at the beginning of the intervention, after controlling the bleeding through direct repair of the artery and placement of introducers, an angiographic check of the ilio–femoral vessels was performed at the end of the intervention. In four cases, an endarterectomy with the placement of a patch on the common femoral artery was necessary; in two cases, an angioplasty with stent placement of the external iliac artery was required due to a dissection presenting a small caliber.

In cases of conversion due to access bleeding after the removal of the introducers, the artery was repaired with the application of a patch. In cases of thrombosis due to dissection of the common femoral artery detected at the end of the intervention, an endarterectomy with patch application was performed. The transfusion was necessary in 21 patients due to low initial hemoglobin levels (Hb < 8 g/dL). All patients undergoing surgical conversion of the access underwent transfusion (*p* < 0.04) with blood loss solely due to failure of the femoral access estimated at around 150 cc. The procedures were mostly performed under local anesthesia with sedation, with one case requiring conversion to general anesthesia following surgical access due to complications from percutaneous access. No perioperative mortality was observed during all procedures, with a mortality rate after 30 days of 16.3%. At the mean follow-up, after 30 days, none of the major complications were observed to be directly connected to the femoral accesses and two lymphoceles were reported as minor complications after the surgical approach. The mean time used for a surgical approach was 7 min (IQR: 5–15); for a percutaneous approach, it was 3 min (IQR: 2–5); and a surgical conversion of percutaneous treatment extended the access time by 12 min (IQR: 5–20). These data are statistically significant. No other statistically significant endpoints were detected using the variables (Table 4).

## 4. Discussion

The repair procedures for AAAs are frequently conducted by a range of specialists, including vascular surgeons, interventional cardiologists, and radiologists. The relative ease of obtaining percutaneous access may encourage practitioners without the necessary surgical skills to opt for this method, reserving surgical intervention primarily for the potential management of complications. However, there are several factors that need to be taken into consideration when selecting the femoral access site for EVAR. These include the size and location of the aneurysm, the presence of calcifications or thrombus in the access vessels, the patency of the iliac arteries, and the availability of suitable access devices. The goal is to choose a site that provides adequate vascular access, minimal trauma to the surrounding tissues, and a straight path to the aneurysm neck. The current literature suggests that percutaneous access to the femoral artery with ultrasound guidance is more advantageous than open surgical repair in the management of aortic aneurysms [13,14]. Only four randomized controlled trials were published comparing two types of access during EVAR, but all trials were judged to have low or very low certainty of evidence with high risk of bias [15,16,17,18].

Buck et al., in a study on 3004 patients undergoing percutaneous access, reported a technical success rate of 96% in the percutaneous EVAR (pEVAR) population and demonstrated significantly shorter hospital stays, shorter operating times, and fewer wound complications [19]. Based on a literature review, the complication rates reported in the literature vary widely depending on the device used, ranging from 4% to 20% [20,21,22]. Among the disadvantages mainly described are embolization, pseudoaneurysms, increases in invasiveness, and postoperative hematomas [23]. Previous cohort studies comparing percutaneous vs. cutdown EVAR have shown that percutaneous access leads to improved outcomes in terms of access site infection, wound healing, and lymphorrhagia/seroma, while it has also been associated with worse outcomes in terms of pseudoaneurysm formation [24].

However, although there are several studies in favor of percutaneous access in the literature, in our experience, this method is useful if there is respect for the anatomical peculiarities of the femoral artery. Pratesi et al., in a retrospective study on 2381 patients, drew attention to calcifications of the femoral artery as a predictive factor of percutaneous access failure [25]. The presence of a hard plaque on the anterior surface of the femoral artery is obviously a problem in percutaneous access due to difficulties in achieving a good puncture and high possibilities of dissection. In the approach with the ProGlide, the presence of calcifications in the anterior wall is known to be a possible factor in the failure of the device. It is thought that calcification in the front may weaken the stitching integrity during closure, which could potentially require additional maneuvers [26,27]. By contrast, MANTA’s closure system could be a more definitive system in these cases; however, the experiences in the literature are not unanimous, and the inability of this type of device to have a backup system requires a more careful selection of patients [28]. Not surprisingly, extensive calcification of the common femoral artery was confirmed as a factor associated with closure failure, especially in determining significant (>50%) arterial stenosis [29]. The development of technologies and devices has led to an expansion of indications for EVAR even outside of the instructions for use (IFUs) [30]. The tortuosities and diameters of the iliac–femoral axes, for example, are important limitations to endovascular treatment [31]. However, increasingly efficient introducers and low-profile devices now allow the overcoming of some obstacles behind which traps may lurk [32]. In these cases, the passage of large caliber introducers can easily damage the iliac arteries with dissections and thrombosis that may compromise the success of the procedure [33]. Such complications can be reduced or better managed by the surgical exposure of the artery. Another limitation to percutaneous access is given by the presence of surgical scars. Bensley et al. reported a diameter of the CFA under 5 mm and a history of previous procedures on the puncture site as predictive factors of failure. They suggest that a small vessel can be treated with percutaneous access through the “dotter” technique, which involves using increasingly larger introducers until the desired caliber for the passage of the final device is reached [34]. It is well-known that a scar represents a significant predictive factor of closure system failure; therefore, where there are issues with percutaneous access, new surgical access can be made, exposing the lower extremity of the iliac external artery or the CFA near the inguinal ligament [35,36,37]. Another problem for percutaneous access can be represented by patients’ obesity. Obesity is a known risk factor for bleeding complications due to the increased difficulty in achieving hemostasis. In obese patients, there is a higher amount of adipose tissue surrounding the femoral artery, making it more challenging to compress the vessel and achieve hemostasis. This can lead to prolonged bleeding at the access site, increasing the risk of hematoma formation and potentially requiring blood transfusions. In these patients, the distance of the puncture site from the artery could be prohibitive for the angiographic needle. Previous studies have shown conflicting results regarding the influence of obesity on pEVAR success rates [38,39,40,41,42]. Access for EVAR remains a challenge in these patients, and not without potential difficulties [43]. In our study, obese patients had a higher rate of surgical conversion after percutaneous access (Table 3) when the CFA depth was greater than 6 cm, while no significant complications were registered with the surgical approach thanks to the simple exposure of the anterior surface of the CFA. In our elective experience, the greater use of percutaneous access is due to the reduction in operating times and the high tolerability of patients. However, in emergencies, a surgical conversion involves an increase in operating times and more time lost in order to control bleeding during rAAAs. These problems were more frequent in the first period of this study and were reduced after the application of the decision-making algorithm, especially in main-body access (Figure 5).

Our data also indicate that damage to the artery is directly linked to the size of the device being used. Therefore, careful consideration must be given to the choice of the side from which to position the main body, making sure it aligns with the quality of access available. Imaging modalities play a crucial role in guiding femoral access during EVAR procedures. Preoperative imaging, such as CT angiography, is essential for assessing the size, location, and morphology of an aneurysm, as well as the condition of the iliac arteries. This information can help the interventional team determine the optimal access site and plan the procedure accordingly [44]. Similar experiences have been reported in the literature. Liang et al. showed a positive experience where the application of a risk model derived from a retrospective study of their own operator data reduced the rate of complications in percutaneous access during EVAR [45]. Furthermore, from the analysis of this study’s data, it was observed that in the initial period, there was no clear criterion for choosing the approach, a choice that was based on the operator’s experience and skill.

While percutaneous access offers advantages in certain cases, it is important for clinicians to carefully evaluate each patient’s anatomical characteristics and medical history before deciding on the approach for femoral artery access [30]. A well-designed algorithm can guide every vascular surgeon through the steps necessary to achieve optimal femoral access quickly and efficiently, reducing the risk of complications and improving the overall success of the procedure.

The standardization of the algorithm will improve the effectiveness of the procedure, making it faster and safer for the patient. In addition, a decision-making algorithm can also be a valuable tool for training purposes, as it provides a structured framework for novice surgeons to follow. By using the algorithm as a guide, trainees can learn the necessary steps for achieving optimal femoral access and gain confidence in their abilities.

While our study has several limitations, including a small patient population, numerous operator-dependent variables that can impact the results, and the inherent constraints of observational study designs, future research and clinical experience will enhance our understanding of the best practices for managing femoral access and aortic aneurysms. Considering the points discussed, our experience should not be viewed as an unequivocal measure of the validity of surgical access in comparison to percutaneous access. However, it certainly serves as a valuable reflection on the practices we engage in daily within the operating room. This ongoing inquiry will help ensure optimal outcomes through the application of appropriate techniques and algorithms.

## 5. Conclusions

Percutaneous access is a minimally invasive method that allows for the rapid improvement of patients, but the length of hospitalization does not differ significantly from the surgical access group. Optimizing femoral access is an essential aspect of successful EVAR procedures, particularly in emergency cases. In our experience, the percutaneous method was a good technique for reducing the rate of complications in surgical femoral access, but surgical conversion occurred more frequently when the anatomic features of the CFA were underestimated. Failed percutaneous access resulted in the longest operative time. DUSs and CT scans were essential for accurately assessing the accesses. A decision-making algorithm for femoral access in emergency EVAR can help streamline the process and improve outcomes by providing a standardized approach to care delivery. By following a systematic algorithm, a vascular surgeon can ensure that all necessary steps are taken to achieve optimal femoral access and minimize the risk of complications.

In this study, the use of a decision-making algorithm helped in decreasing complications and operating time, with fewer surgical conversions required during the procedure.

## Figures and Tables

**Table 1:** Nonanatomic patient variables.

		N (%)
Diabetes		54 (73)
Hypertension		70 (96)
Tobacco Use		64 (86)
Renal Failure		23 (31)
Obesity		
	Obesity Grade I (BMI 30–34.9)	5 (6.7)
	Obesity Grade II (BMI 35–39.9)	6 (8.1)
	Obesity Grade III (BMI > 40)	3 (4.1)

**Table 2:** Medical therapy before intervention.

Antiplatelet Therapy			N %
	None		25 (33.8)
	Single Agent		45 (60.8)
		Acetylsalicylic, 100 mg	31
		Acetylsalicylic, 150 mg	5
		Acetylsalicylic, 300 mg	3
		Clopidogrel	9
		Ticlopidine	1
	Dual Therapy (Acetylsalicylic 100 mg + clopidogrel)		0
Anticoagulation			
	Warfarin		3 (4.1)
	Direct Oral Anticoagulants		1 (1.3)

**Table 3:** Surgical conversion after percutaneous access (74 patients—148 accesses).

Group A: 46 Patients	Surgical Approach	Percutaneous Approach	Conversion	*p*
N. accesses	40	52	10	
Severe atherosclerosis with calcifications of >50% of circumference	8	26	3	0.5
Small CFA (<5 mm)		8	2	0.4
Obesity patients: BMI > 35		7	4	0.5
Tortuosity of iliac–femoral axis: >1.6		11	1	0.6
**Group B:** **28 patients**			1	
N. accesses	39	17		
Severe atherosclerosis with calcifications of >50% of circumference	19	0		0.5
Small CFA (<5 mm)	10	0		0.7
Obesity patients: BMI > 35	2	0		0.5
Tortuosity of iliac–femoral axis: >1.6	8	5	1	0.2

**Table 4:** Operative details.

Variables	Surgical	Percutaneous	Conversion	*p*
Operative time, min [IQR]	79 (68–85)	66 (60–75)	85 (78–122)	
Rate of transfusion (21 patients—28.4%)	12 (57%)	9 (43%)	11 (100%)	<0.04
Type of anesthesia	Local: 42, general: 3	Local: 28, general: 1	Local: 10, general: 1	
CFA approach time, min [IQR]	7 (5–15)	3 (1–5)	12 (5–20)	<0.04
Mean contrast medium, mL [IQR]	80 (75–91)	81 (77–93)		
Fluoroscopy time, min [IQR]	20 (11–21)	21 (13–23)		
DAP, G * cm^2^ [IQR]	30 (27.5–33)	32 (28–36)		
**Implanted Endoprostheses:** **74 cases**				
Endurant (Medtronic)	36	26	8	
Treo (Bolton)	3	1	0	
AFX (Endologix)	6	2	1	

DAP: dose area product.

## Data Availability

In appropriate cases, data can be requested from the corresponding author.
